# Hemophagocytic Lymphohistiocytosis and Clostridium difficile Infection: A Diagnostic Dilemma

**DOI:** 10.7759/cureus.33865

**Published:** 2023-01-17

**Authors:** Kimberly Boldig, Amy Kiamos, Avni Agrawal, Pramod Reddy

**Affiliations:** 1 Internal Medicine, University of Florida College of Medicine – Jacksonville, Jacksonville, USA; 2 Internal medicine, University of Florida College of Medicine – Jacksonville, Jacksonville, USA

**Keywords:** immune system dysregulation, hyperferritinemia, septicemia, clostridium difficle infection, hemophagocytic lymphohistiocytosis (hlh)

## Abstract

Hemophagocytic lymphohistiocytosis (HLH) is a life-threatening condition that often goes underdiagnosed because of broad and non-specific symptomatology, usually consisting of fever, hepatosplenomegaly, and multiorgan failure. This disorder can be driven by genetic components (primary) or acquired (secondary) causes related to infectious, autoimmune, or malignant processes. HLH pathogenesis derives from overactive and dysregulated immune system responses. This disorder often goes misdiagnosed because of similar clinical and laboratory findings to septicemia. Cases of HLH most commonly coexist with Epstein-Barr virus (EBV). *Clostridium difficile (C. difficile) *infection causing HLH has also rarely been described in the literature. A firm knowledge of HLH association with clostridial infection is essential to recognize. A presumed diagnosis of HLH in a decompensating patient may prompt the initiation of appropriate treatment earlier and improve clinical outcomes. We discuss the diagnostic and management difficulties associated with these concurrent conditions.

## Introduction

Hemophagocytic lymphohistiocytosis (HLH) is a condition that causes dysregulation of the immune system and typically affects children [1]. Pathogenesis of HLH relates to uncontrolled histiocytes and lymphocyte recruitment, hypersecretion of cytokines, tissue infiltration, and multiorgan failure [2]. When seen in children, it is often associated with a genetic predisposition known as familial or primary HLH. Several genes are associated with HLH including PRF1, UNC13D, STXBP2, STX11, MUNC13-4, granule/pigment abnormality genes (RAB27A, LYST, and AP3B1), X-linked lymphoproliferative disease genes (SH2D1A and XIAP), NLRC4, and CDC42 [3,4]. Other genetic predispositions to HLH include primary immune deficiencies, inborn errors of metabolism, and rheumatologic or autoinflammatory disorders [4]. HLH is also associated with malignancy and infection [3,4]. Acquired or secondary HLH is most often seen in adults and is associated with autoimmune, infectious, immunosuppressive, or malignant conditions [1-3]. Data on the incidence of HLH is limited. Primary HLH is believed to occur in one in 30,000-50,000 births in Sweden [2]. In Japan, the incidence has been reported at 1 per 800,000 [5].

HLH has a high mortality rate likely associated with diagnostic delays leading to delays in treatment. Without therapy, active familial HLH survival is about two months [6,7]. Untreated acquired HLH is associated with survival of a few months, and overall mortality is 41-75% [3]. Diagnosis of HLH is based on guidelines set forth by the Histiocyte Society (HLH-94 and HLH-2004) based on clinical presentation and laboratory values [7]. Diagnostic criteria consist of fever, splenomegaly, bicytopenia, hypertriglyceridemia, hypofibrinogenemia, hemophagocytosis, low/absent NK-cell-activity, hyperferritinemia, and high-soluble interleukin-2-receptor levels (sCD25). Five of the eight criteria must be met to confirm the diagnosis of HLH [3,4,7]. Patients often present with hepatitis, coagulopathy, liver failure, central nervous system involvement, and multiorgan failure. Diagnosis and treatment of HLH can present a challenge for clinicians as the presentation may be confused for septicemia.

Secondary HLH has been associated with various infectious etiologies. Epstein-Barr virus (EBV) is the most common viral infection associated with the acquisition of HLH. EBV typically infects B cells, while cytotoxic T cells respond and regulate the infected B cells. In HLH, EBV infects T cells causing clonal proliferation and producing the cytokine storm associated with pathogenesis [2,3]. Other infectious causes of HLH include cytomegalovirus (CMV), parvovirus, herpes simplex virus, norovirus, varicella-zoster virus, measles virus, human herpes virus 8, H1N1 influenza virus, HIV, Mycobacterium tuberculosis, rickettsia, Escherichia coli, leishmania, and histoplasmosis [3]. We present a complicated case of HLH and *Clostridium difficile* (*C. difficile*) infection, an association that is rarely described in the literature.

## Case presentation

A 29-year-old female with a past medical history of HLH presented to the hospital with nausea, vomiting, and non-bloody diarrhea. She was diagnosed with HLH at an outside hospital and had received prior treatment for HLH with intravenous immunoglobulin (IVIG), dexamethasone, and etoposide. There was a plan for intrathecal methotrexate; however, she developed transaminitis with associated lower extremity swelling and pain, causing a delay in treatment. She was subsequently lost to follow up until presentation to our hospital.

**Figure 1:**
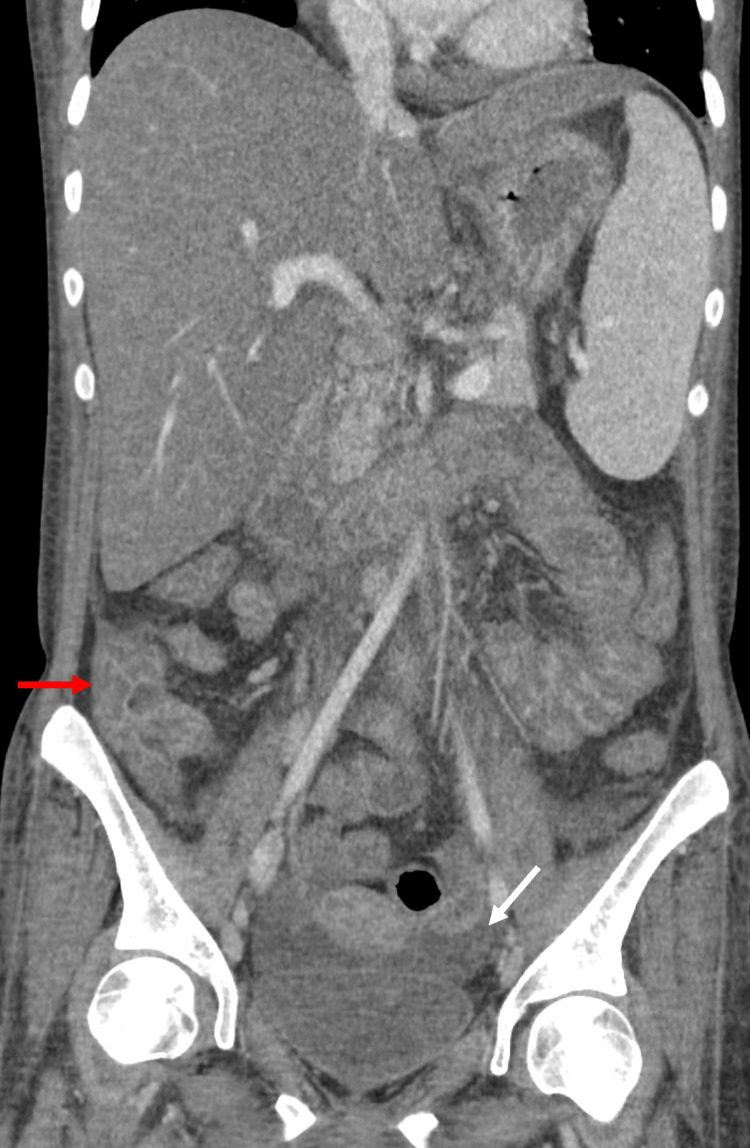
CT abdomen and pelvis coronal reformatted image demonstrating hepatosplenomegaly, diffuse colonic wall edema most pronounced in the cecum (red arrow), and pelvic free fluid (white arrow).

Upon presentation to our institution, she was febrile, hypotensive, and tachycardic. Laboratory workup was significant for leukocytosis and microcytic anemia (Table 1). She had elevated ferritin that was persistent compared to previous values. At this presentation, the ferritin level was 13,858. She was tested at presentation for *C. difficile* infection because of her immunocompromised state in the setting of chronic steroid use. Stool nucleic acid amplification testing for *C. difficile* infection was positive, and she was immediately started on oral vancomycin. A CT scan of the abdomen and pelvis demonstrated pan-colonic bowel wall mural thickening, edema, hepatosplenomegaly, and small mesenteric and periportal lymph nodes (Figure 1). The patient later developed hemodynamic instability, and her antibiotics were switched to a broad-spectrum regimen.

**Table 1:** Laboratory values at presentation.

	WBC (10E3/uL)	Hemoglobin (g/dL)	Mean Corpuscular Volume (fl)	Ferritin
Reference range	4.5-11	12-16	82-101	15-150 ng/ml
Value	19.28	10.9	72.7	13,858

Given her previous HLH diagnosis and decompensated clinical status, she was started on IVIG and steroids. The patient required intubation, and she was noted to subsequently develop diffusely scattered petechiae. She was found to have severe anemia and thrombocytopenia that persisted despite blood transfusion and platelets. Coagulation labs returned suggestive of severe disseminated intravascular coagulation (DIC). The patient decompensated further and expired.

## Discussion

HLH is a complex disease with broad pathogenesis. Although the pathogenesis of primary or acquired HLH may differ, diagnostic criteria remain consistent. HLH is often underdiagnosed or diagnosed late due to broad and nonspecific clinical presentations. Some patients may have atypical presentations or may not meet the requirement of 5 out of 8 criteria. In these adult patients, genetic analysis may assist with further diagnostic and treatment decisions [3,4]. Delays in laboratory studies can also further complicate the diagnostic picture [3]. Despite disease nomenclature, hemophagocytosis seen on histopathology of bone marrow or cellular tissue is a poor diagnostic clue for the disease. Hemophagocytosis is represented by the phagocytosis of erythrocytes, leukocytes, platelets, and precursor cells by histocytes [3]. Hemophagocytosis has poor sensitivity and specificity and is ultimately considered one of the least important diagnostic criteria [3,4,6,8].

Having high clinical suspicion for diagnosis is important to allow proper initiation of treatment. Treatment is based on HLH-94 or -2004 protocol targeting the hyperinflammatory state with cytotoxic and immunosuppressive therapy to destroy cytotoxic T lymphocytes and macrophages. Treatment consists of chemo-immunotherapy. Hematopoietic stem cell transplant is recommended in patients with central nervous system involvement, genetic, or severe disease [3,6,7]. Some patients are unresponsive to initial therapy, resulting in refractory disease and a dismal prognosis [6,9]. The relapsed disease may be identified through transaminitis, cytopenias, elevated ferritin, sCD25, or sCD163, a marker of macrophage activation [3,6]. Relapse is treated with the reintroduction of initial therapy [6]. New treatment options for the relapsing or refractory disease include emapalumab, a monoclonal antibody that inhibits interferon-γ (IFN-γ) modulating cytokine overproduction [10,11].

HLH presentation is frequently confused for an infectious or septic clinical presentation. Both presentations are characterized by hyperinflammation, fever, and deteriorating clinical pictures [12]. Additionally, the infection may trigger secondary HLH, further complicating the diagnosis. Efforts to differentiate the two disease states have identified ferritin as an important diagnostic marker. Ferritin levels greater than 500 ng/ml should raise suspicion for HLH; however, levels above 7,500-10,000 ng/ml are more specific [12]. Adding to the clinical complexity, treatment for these two diseases is vastly different. Initiating immunosuppressive therapy in the setting of sepsis in a patient with HLH is a difficult treatment decision. Sepsis without an underlying condition, such as HLH, is more common clinically, and immunosuppressive therapy, in this case, could be detrimental to patient outcomes. Treating the underlying bacterial, viral, or fungal cause is critical to HLH management. It is important to initiate HLH treatment despite unresolved infection, cytopenias, or organ dysfunction [3,6]. Studies have shown that some patients respond to the targeted infectious treatment and ultimately avoid HLH chemotherapy [3]. It has been proposed to start treatment with short-term corticosteroids if an HLH diagnosis is unclear and to increase steroid doses once confirmation is made [12]. Intravenous immunoglobulins (IVIG) may also be used for immune modulation when the diagnosis is unclear [12].

*C. difficile* is usually associated with antibiotic use, older age, or recent hospitalization. However, 5% of adults are said to be colonized with this bacteria [13]. The clinical presentation of *C. difficile* can be broad, ranging from mild diarrhea to fulminant colitis. Complications include toxic megacolon, colonic perforation, kidney failure, septic shock, bacteremia, and death [13]. The association of *C. difficile* and HLH is rare. One case report described a patient with EBV-positive natural killer T (NKT) cell lymphoma who developed a *C. difficile* infection [14]. The patient developed fevers, transaminitis, leucopenia, thrombocytopenia, bacteremia, delirium, hypotension, and respiratory distress. The patient eventually passed away and was diagnosed with HLH from autopsy results [14]. A second case report described a patient with ulcerative colitis (UC) undergoing treatment with 6-mercaptopurine (6-MP) who was found to have CMV and *C. difficile* infection. A bone marrow biopsy was performed due to indicative labs demonstrating hemophagocytosis. The patient passed away despite treatment with etoposide and steroids [15].

Our case presentation represents the diagnostic difficulties associated with identifying an HLH flare versus sepsis secondary to *C. difficile* infection. Upon initial presentation, our patient did not present with cytopenias typically seen in HLH. Ferritin was checked three days into admission and was found to be significantly elevated; however, it was persistent compared to previous values. This brings to question the ferritin trend seen in a patient with previous relapsing HLH. There is very limited data about ferritin trends in relapsing or treated HLH. It has been argued that a greater decrease in ferritin levels after treatment initiation may be predictive of treatment outcomes and improved survival [16,17]. Our patient's persistently elevated ferritin further complicates the diagnostic picture in identifying an HLH flare. Hypofibrinogenemia was identified in our patient; however, it was identified in the setting of DIC, limiting its utility to the diagnosis of HLH. Assessing for hemophagocytosis, NK cell activity and sCD25 concentration were not evaluated due to the patient's rapid decline. The clinical course of the patient remained complicated as she was refractory to broad-spectrum antibiotics, steroids, and IVIG.

## Conclusions

Our case and review of the literature demonstrate the importance of prompt diagnosis and initiation of treatment in HLH. In our case and the two cases reviewed, all three patients quickly decompensated in the setting of coexisting diseases. We suspect there have been more patients with a concurring diagnosis of HLH and *C. difficile*. However, because of the similar presentation to a septic presentation, further diagnostics may not take place, contributing to the underdiagnosis of HLH. It is also important to note that our patient had a previous diagnosis of HLH but was lost to follow-up outpatient. Close outpatient follow-up is essential to optimize patient survival and could have improved the clinical outcome in our case. The health disparity associated with our case presentation may be a notable contributor to our patient's morbidity and mortality, as our patient was not on optimal therapy for her diagnosis.
